# Editorial: Multidisciplinary Approaches in Exploring Cancer Heterogeneity, TME and Therapy Resistance: Perspectives for Systems Medicine

**DOI:** 10.3389/fcell.2022.842596

**Published:** 2022-02-03

**Authors:** Brigitte M. Pützer, Kanaga Sabapathy

**Affiliations:** ^1^ Institute of Experimental Gene Therapy and Cancer Research, Rostock University Medical Center, Rostock, Germany; ^2^ Department Life, Light & Matter, University of Rostock, Rostock, Germany; ^3^ Division of Cellular and Molecular Research, Humphrey Oei Institute of Cancer Research, National Cancer Centre Singapore, Singapore, Singapore; ^4^ Cancer and Stem Cell Biology Program, Duke-NUS Medical School, Singapore, Singapore

**Keywords:** cancer heterogeneity, tumor immune microenvironment, development and homeostasis, cancer evolution, p53 family transcription factors, phenotypic plasticity, computational methods and data mining, biomarkers and personalized therapeutics

Despite significant advances that have shattered previous dogmas about the causes of tumor metastasis, the development of therapies to treat or prevent aggressive disease progression has not kept pace and remains the most important challenge. Cancer heterogeneity, to a large extent accounting for the incomplete and temporary efficacy of current anticancer measures, is still poorly understood at the molecular level. While early tumor stages are shaped by the accumulation of driver mutations, advanced cancers have a number of key adaptations or hallmarks that can contribute to metastasis (Birkbak and McGranahan, 2020).

Coherences between epithelial-mesenchymal transition (EMT) and the emergence of cancer stem cells highlight that the metastatic process is driven by epigenetic programming that involves short and long non-coding RNAs (Meier et al., 2016; Wang et al., 2016; Logotheti et al., 2020a). These events are usually cell- or tissue-specific and regulated at different developmental stages or in response to extracellular stimuli (Vanharanta and Massagué, 2013; Khan et al., 2017). Furthermore, combinatorial *de novo* activation of multiple distinct and developmentally distant transcriptional modules appears to be a recurrent mechanistic pattern (Rodrigues et al., 2018). In this regard, co-option of programs of tissue homeostasis and normal embryonic development, including off-context expression of tissue-restricted genes or reactivation of cell differentiation pathways in the cancer context (Logotheti et al., 2020b) emerge as predictors of poor patient outcome across various cancers. Another layer of heterogeneity and complexity that promotes disease progression arises from reciprocal cross-talks of cancer cell subpopulations with cellular and molecular components of the tumor microenvironment (TME) which massively influences the treatability of metastasis-prone cancer cells.

The p53 family of transcription factors (p53, p63, p73) that includes tumor suppressor proteins and their N-terminally truncated or mutant isoforms, is critically important for orchestrating the above processes. They cover a wide range of non-oncogenic and oncogenic functions by switching duties depending on the cellular and molecular background (Vikhanskaya et al., 2007; Crum and McKeon 2010; Toh et al., 2010; Steder et al., 2013; Vera et al., 2013; Engelmann and Pützer 2014; Dulloo et al., 2015; Engelmann et al., 2015; Nemajerova et al., 2018; Melino, 2020; Wang et al., 2020; Rozenberg et al., 2021).

This Research Topic creates a conceptual framework for systems medicine approaches using information from multiple disciplines, such as developmental biology, cancer research and tumor immunology, to understand disease phenotypes based on common mechanisms and in an integrative manner. A total of 11 articles were received, of which 6 are original research and 5 are review articles.

Based on latest achievements in the field, suggesting that cancer acquires metastatic potential and evolves via co-opting gene regulatory networks of embryonic development and tissue homeostasis frequently conserved among species, Marquardt et al. focused on tumor evolution, specifically on metastatic potential in relation to organismal evolution. The authors analyzed the first appearance of tumors and the transition between non-metastatic and metastatic tumors during the evolution of phylogenetic taxa using bioinformatic tools in species-specific cancer phenotypes, multi-omics data, developmental phenotypes of knockout mice, and molecular phylogenetics. This systems-based approach provides evidence that the presence of metastasis coincides with agnatha-to-gnathostome transition, and that genes indispensable for jaw development are co-opted in tumor progression. The in-silico pipeline developed here enables prediction of putative metastatic drivers and targeting of evolutionary traits in the evolving tumor.

The relevance of lncRNAs in competing endogenous RNA (ceRNA) mechanisms and cancer regulatory networks is addressed by Zhang et al. This study highlights the effects of lncRNA somatic mutations in miRNA response elements on the expression of target mRNAs (ceM) and how this affects tumor heterogeneity. Multivariate multiple regression models showed a significant effect of 162 high-frequency mutations on the expression of ceMs and low-frequency mutations resulted in perturbation of 1624 ceMs in pan-cancer. The authors provide data underlining the impact of lncRNA mutations on changes in oncogenic functions and patient survival.

Other excellent contributions investigate context-specific mechanisms of treatment resistance, with emphasis on immunotherapy to define markers for improved responses and clinical need in different cancer settings but mainly melanoma. Considering the potentially essential role of tumor-associated B (TAB) cells in T cell-based anti-tumor immunity, Chen et al. explored the developmental changes of B cells during melanoma progression. By using seven color multiplex immunohistochemistry and automated tissue imaging, the authors analyzed the six major B cell and antibody secreting cell (ASC) subpopulations and their spatiotemporal dynamics in whole tumor sections of a large set of human melanoma samples. Their data point to a metastasis-, tumor stage-, and age-associated distribution of subpopulations with decreased memory-like TAB in metastasizing primary melanomas, but increased numbers at locoregional metastatic sites, and an enrichment for plasmablast and plasma cell-like ASC at distant metastatic sites.

The work of Lai et al. is dedicated to the improvement of dentritic cell (DC)-based vaccines in the tumor microenvironment. Authors constructed a multi-compartment Ordinary Differential Equation model representing different stages of DC immunotherapy, such as spreading and bio-distribution of intravenously injected DCs, biochemical reactions regulating DC maturation and activation, and DC-mediated T cell activation to analyze DC- and T cell-associated molecules and signaling pathway predicting the optimal targets for enhancing DC bioactivity and melanoma-specific cell therapy. Their key finding is that modulating the NF-kB inhibitor IκBα may improve differentiation of memory T (Tmem) cells.

Toy et al. uncover molecular markers of cancer radioresistance based on high-throughput gene expression data. They applied a bioinformatics approach using different methods and computational pipelines to publicly available transcriptome datasets. Results show a set of 36 differentially expressed genes primarily linked to DNA damage repair, oxidative stress, and apoptosis in common radioresistant-relevant pathways. These findings and their value as potential diagnostic markers or therapeutic targets can be validated by *in vivo* experimental studies to improve treatment outcomes.

Furthermore, several cutting-edge review articles provide an updated overview of the roles of p73, p53 and p63 as key drivers of phenotypic and functional plasticity in the context of cellular reprogramming, tissue remodeling and cancer progression, connecting intracellular events with complex and dynamic microenvironments. Focusing on published genome-wide studies, Woodstock et al. outline recent findings of a cooperative, instead of the originally known, competing interplay between p53 and Δp63, and explore how p53 family members that share common binding sites and target genes coordinate their effects on cell fate.

Laubach et al. highlight the impact of non-canonical functions of p53 family proteins in a plethora of biological processes, and refer specifically to studies that demonstrate the roles of p53, p63, and p73 in lipid and iron metabolism. Lipids are important for many cellular functions including structure, signaling, and the inflammatory response, as pointed out by recent publications. Authors discuss the similarities and differences of all three proteins in regulating these metabolic processes and their relevance to disease.

The function of p73 beyond its well-established tumor suppression effect is comprehensively addressed in the review of Maeso-Alonso et al. They summarize latest evidence for the role of p73 as a tissue architect that governs the organization and homeostasis of different microenvironments, supporting processes like multiciliogenesis, hippocampal neurogenesis, and spermatid development. This function is considered to be a conserved trait inherited from the p63/p73 hybrid-like gene ancestor at the beginning of epithelial tissue evolution tracing back to Placozoans and Cnidaria. Via integration of ChIP- and RNA-seq data, studies analyzed are further linked to their own data on p73-mediated regulation of cytoskeletal dynamics, corroborating their hypothesis.

Focusing on the structure and variegated functions of p73 isoforms, the work of Logotheti et al. characterizes the significance of TP73 in controlling development and differentiation, and how this activity can be hijacked during cancer progression or in the tumor microenvironment, with emphasis on neoneurogenesis as emerging cancer hallmark. Using melanoma as a paradigm, they provide new insight into molecular mechanisms underlying the pleiotropic effects of p73 based on the nature of p73 isoforms, the presence of interactors, the architecture of target promoters, and subcellular localization. The authors envision that dysregulation of one or more of these parameters in tumors promote aggressive metastatic stages by reactivating p73 isoforms and/or p73-regulated differentiation programs, in a spatiotemporally inappropriate manner.

Interdisciplinary work and the combination of wet- and dry-lab skills are ideal requirements for future translational research. The contributions collected in this Research Topic provide deeper insights into cancer etiology, molecular mechanisms, heterogeneity, and the role of the tumor microenvironment in metastasis. This will influence the development of individualized next-generation cancer therapeutics. Moreover, advances in biomaterial and 3D cell culture technologies like spheroids, organoids, and organs-on-chip techniques are opening new opportunities for testing patient-specific therapies.
